# Pesticides in Formulations: New Revolutionary Findings

**DOI:** 10.3390/toxics12020151

**Published:** 2024-02-15

**Authors:** Gilles-Eric Seralini

**Affiliations:** Network on Risks, Quality and Sustainable Environment and Faculty of Sciences, University of Caen Normandy, 14032 Caen, France; seralini.gilles-eric@neuf.fr

Everything began with the discovery that pesticides have long had unintended side effects on non-target species, which is illustrated by Ponepal et al. in this Special Issue (contribution 1). In fact, pesticides, whether used in agriculture or indoors, are always sold and used as mixtures, called formulations, which are not single chemicals but always contain adjuvants; however, their nature is not fully detailed. However, the negative unintended effects of these mixtures are thus largely demonstrated in the whole ecosystem for numerous formulations because they are the only ones used there, and there are many. It is true that species are not only exposed to the entirety of a single commercial formulation in nature but also to mixtures of a wide range of residual pollutants including, of course, pesticides but also plasticizers of different sizes, such as nanoparticles (some of which are incidentally used in adjuvants), as well as various heavy metals or metalloids, additives, preservatives, petroleum molecules, etc. The very stable residues of our industrial chemicals are all present and have considerably increased to reach any form of life in a chronic way since pollutants are spread everywhere. This was the intent of the interesting, real-life risk simulation conducted by Vardakas et al. in this Special Issue (contribution 2); this method should be used more regularly, as demonstrated and explained by the authors and detailed in the study protocol by Karzi et al. in this Special Issue (contribution 3). Numerous chemicals, such as bisphenols and phthalates, are used as pesticide adjuvants, but they are also used as plasticizers. This is the crucial meaning of studying the real-life effects of mixtures to which we are truly exposed. Their real long-term effect is not only the additive theoretical effect, as demonstrated in this Special Issue, and could explain numerous chronic human or animal diseases in various kingdoms or even in plants or microbes where there is also a loss of biodiversity.

But let us first study commercially proposed pesticide formulations because they request regulatory authorizations to be assessed. It is then a scientific surprise for the profession of toxicology to observe that toxicity cannot be deduced by industries and authorities using declared purified ingredient toxicity studies, given known classical admissible daily intake (ADI) values plus some theoretical, scientifically unclear considerations of the adjuvants in model formulations. Obviously, underestimation occurs. Most researchers base their reasoning, as per usual, on these data, which are found in the literature. The formulations are in fact much more potent with respect to side effects since these are mixtures designed to act on whole organisms, to penetrate plant or insect cuticles first or to enter various membranes, impacting fungi and bacteria, whether symbiotic, such as in gut microbiota, or pathogenic; this cannot occur through a specific uniform mechanism. They act on human cells differently than declared ingredients do, as was perfectly confirmed in eight cases by Ferguson et al., 2022, in this Special Issue (contribution 4). This was previously established in 2005 by Richard et al. [1], and it was confirmed and extended to various models in 2007 and 2009 [2,3] and especially with more precision in 2013 and 2014 by Mesnage et al. [4,5]. In fact, mass spectrometry analyses of mixtures of pure, usable, commercially available formulations of pesticides revealed hundreds of unidentified compounds spread over Gauss curves [4], indicating that adjuvants are not only variable and numerous but also contain residual chemicals. More importantly, these make mixtures hundreds to thousands of times more efficient with respect to oxidative stress in vivo and in patients, as demonstrated in court during the Monsanto Affair, or in human placentas [6] than what is expected from the sole ADI of the purified declared ingredient in the long term that serves as basic data. This insufficiency in assessing chronic effects was confirmed by detailed omics studies in vivo [7,8].

It is not only a matter of the solubility of the declared ingredient in combination with a list of numerous, unknown, known, declared, and undeclared adjuvants; the entirety of many formulations containing the same declared ingredient are thus composed of formulants. This has been checked out. The adjuvants or formulants have their own unassessed toxicities in mixtures or by themselves, as determined via long-term tests by industries, authorities, or international safety agencies. They could be called co-formulants of the main ingredient, but that is true only if the declared ingredient was really the main and most active (toxic) one, but this is not known due to several uncertainties regarding pesticide compositions, not all are declared and known by authorities; in addition, because of industrial secrets and permanent changes in formulations and lots in commercialization. This has been demonstrated, even in plants, for the famous herbicide glyphosate, which is not the most active among glyphosate-based herbicides [9]. Heavy metals and metalloids present in petroleum, such as arsenic, nickel, and lead, are in fact in adjuvants of pesticides [9], which act in combined forms even at low levels.

What are the other usual compounds in pesticide formulants? They are very clearly demonstrated, characterized, and identified for recent compounds in this Special Issue by Jungers et al. (contribution 5). They are residues of petroleum, which have been known for their pesticidal activity since 1787 and for their toxicity and carcinogenicity since 1953 (contribution 5)! It is no surprise, then, if they are not declared, and if their real toxic effects are falsified by industry, as demonstrated by Novotny in this Special Issue (contribution 6), and/or by national or international authorities, as demonstrated by Bacon et al., also in this Special Issue (contribution 7). Condemnations and demonstrations of this occurred in court in the USA for the glyphosate-based pesticide affairs, which involved hundreds of thousands of patients at least, in the Monsanto Affair [10], and mixtures of plasticizer PCBs (which can also be present in the adjuvants of some pesticides) by the same industry, belonging to Bayer, for which toxicity was also hidden [11].

The aim of this Special Issue is thus to explain new discoveries, particularly regarding the long-term toxicities of pesticides in whole ecosystems, and, importantly, how these are taken into consideration by health and regulatory agencies. The scope is also to understand new developments in the knowledge of the toxicity of full formulations, especially compared to the known effects of the declared active ingredients purified alone, which thus cannot serve be used in a basic approach.

Finally, this Special Issue demonstrates that the full formulations of pesticides must be experimentally tested at a toxicological level, especially for long-term or chronic effects. This has never been requested by authorities, even for models of commercial formulations. However, the formulations and their components enter food, feed, and the ecosystem, where they are not assessed. The major pesticides in the world, i.e., glyphosate-based herbicides, which are being debated on all continents, represent one of the first problems but not the only problem. Neonicotinoids and others can be evoked.

This knowledge is crucial for shedding light on the real toxicity of pesticides. Because of major toxicological differences between declared ingredients and commercialized formulated pesticides, which are largely underestimated today, and because long-term toxicity is never really studied, tested experimentally in vivo, or requested by regulatory authorities, even once for model formulations, there is a crucial scientific gap at this level. Also, because the toxicity of a pesticide cannot be anticipated or deduced from the known toxicity of the sole declared ingredient (e.g., the ADI), plus additional theoretical calculations or the toxicity thresholds of unknown or undeclared adjuvants, these factors are currently ignored by scientists; thus, this Special Issue will shed new light on the necessary revolution in toxicology. Thresholds of toxicity should be decreased by several hundreds or thousands of times below ADI values, again, due to undeclared, untested existing mixtures in pesticide formulations.

## References

[1] Richard S., Moslemi S., Sipahutar H., Benachour N., Seralini G.-E. (2005). Differential effects of glyphosate and roundup on human placental cells and aromatase. Environ. Health Perspect..

[2] Benachour N., Sipahutar H., Moslemi S., Gasnier C., Travert C., Seralini G.E. (2007). Time- and dose-dependent effects of Roundup on human embryonic and placental cells. Arch. Environ. Contam. Toxicol..

[3] Benachour N., Séralini G.-E. (2009). Glyphosate formulations induce apoptosis and necrosis in human umbilical, embryonic, and placental cells. Chem. Res. Toxicol..

[4] Mesnage R., Bernay B., Séralini G.-E. (2013). Ethoxylated adjuvants of glyphosate-based herbicides are active principles of human cell toxicity. Toxicology.

[5] Mesnage R., Defarge N., Spiroux de Vendômois J., Séralini G.-E. (2014). Major pesticides are more toxic to human cells than their declared active principles. BioMed Res. Int..

[6] Simasotchi C., Chissey A., Jungers G., Fournier T., Seralini G.E., Gil S. (2021). A glyphosate-based formulation but not glyphosate alone alters human placental integrity. Toxics.

[7] Mesnage R., Arno M., Costanzo M., Malatesta M., Séralini G.E., Antoniou M.N. (2015). Transcriptome profile analysis reflects rat liver and kidney damage following chronic ultra-low dose Roundup exposure. Environ. Health.

[8] Mesnage R., Renney G., Séralini G.E., Ward M., Antoniou M.N. (2017). Multiomics reveal non-alcoholic fatty liver disease in rats following chronic exposure to an ultra-low dose of Roundup herbicide. Sci. Rep..

[9] Defarge N., De Vendômois J.S., Séralini G.E. (2018). Toxicity of formulants and heavy metals in glyphosate-based herbicides and other pesticides. Toxicol. Rep..

[10] Seralini G.E., Douzelet J. (2020). The Monsanto Papers.

[11] Rosner D., Markowitz G. (2023). “Ashamed” to Put His Name to It: Monsanto, Industrial Bio-Test Laboratories, and the Use of Fraudulent Science, 1969–1985. Am. J. Public Health.

